# Comparative efficacy of platelet-rich plasma (PRP) injection versus PRP combined with vitamin C injection for partial-thickness rotator cuff tears: a randomized controlled trial

**DOI:** 10.1186/s13018-024-04917-3

**Published:** 2024-07-23

**Authors:** Fatemeh Mohammadivahedi, Amirreza Sadeghifar, Alireza Farsinejad, Sara Jambarsang, Hamid Mirhosseini

**Affiliations:** 1 Master Science Student of Surgical Technology, Shahid Sadoughi University of Medical Sciences, Yazd, Iran; 2 Student Research Committee, Shahid Sadoughi University of Medical Sciences, Yazd, Iran; 3 Department of Orthopedics, School of Medicine, Kerman University of Medical Sciences, Kerman, Iran; 4 Stem Cells and Regenerative Medicine Innovation Center, Kerman University of Medical Sciences, Kerman, Iran; 5 Departments of Biostatistics and Epidemiology, School of Public Health, Center for Healthcare Data Modeling, Shahid Sadoughi University of Medical Sciences, Yazd, Iran; 6 Research Center of Addiction and Behavioral Sciences, Shahid Sadoughi University of Medical Sciences, Yazd, Iran; 7 Department of Operating Room and Anesthesiology, School of Allied Medical Sciences, Shahid Sadoughi University of Medical Sciences, Yazd, Iran

**Keywords:** Rotator cuff tears, Platelet-rich plasma, Vitamin C, Partial-thickness rotator cuff tears, Ultrasound guided injection, Ascorbic acid, PRP

## Abstract

**Background:**

The optimal approach for managing partial-thickness rotator cuff tears (PTRCT) remains controversial. Recent studies related to PTRCTs have shown that platelet-rich plasma (PRP) injection might be an effective treatment option. Despite the role of vitamin C in collagen synthesis and its antioxidant properties, the effects of combined PRP and vitamin C treatment on rotator cuff repair are not well understood. This study investigated the effect of combined treatment of PRP and vitamin C treatment on PTRCTs.

**Methods:**

One hundred-ten patients with PTRCTs were randomly allocated to two groups and underwent subacromial injections of either (A) normal saline and platelet-rich plasma or (B) vitamin C and platelet-rich plasma. The Constant score, American Shoulder and Elbow Surgeons (ASES) score, and visual analog scale were used to evaluate the outcomes before, 1 month after, and 3 months after injection.

**Results:**

At the 3-month follow-up, no statistically significant differences were observed between the two groups in terms of ASES and Constant scores. Although a slight difference favoring group B was noted in functional scores and pain reduction, this difference was not statistically significant. However, both groups demonstrated significant pain reduction over time (*p*-value < 0.001). Additionally, the enhancement of ASES and Constant scores in both groups was statistically significant (*p*-value < 0.001).

**Conclusions:**

In conclusion, both PRP injection alone and PRP combined with vitamin C led to significant reductions in pain and enhancements in function scores over time (*p* < 0.001), suggesting the effectiveness of PRP as a non-surgical treatment for PTRCTs within 3 months. While PRP alone showed significant benefits, further research is required to ascertain if the combination therapy offers statistically significant advantages over PRP alone.

**Trial registration:**

Clinical trial registration code: IRCT20230821059205N1.

## Background

Rotator cuff tears (RCTs), which account for up to 40% of shoulder joint diseases, are associated with pain and limitations in physical activity [1, 2]. Based on the size of the tear, RCTs can be classified as a full-thickness tear or a partial-thickness tear [3].

The surgical management of RCTs is a therapeutic choice for younger individuals experiencing acute symptomatic partial or full-thickness tears with significant functional impairment. Conversely, conservative management is frequently employed in individuals exhibiting tendon degeneration or disruptions affecting less than 50% of the total tendon thickness [4–7]. Considering the surgical risks and the significant risk of retear after repair, conservative approaches are typically prioritized as the initial treatment option, particularly among older populations [8]. These conservative interventions include physiotherapy, activity modification, painkillers, nonsteroidal anti-inflammatory drugs (NSAIDs), and corticosteroid injections. Moreover, their efficacy in halting the progression of the illness is frequently limited [9–11].

In recent times, there has been a surge of interest in the mechanisms of biological treatment to improve pain remission and enhance the function of injured tendons and muscles. An autologous blood product known as platelet-rich plasma (PRP) is centrifuged from one’s own blood with a high concentration of platelets [12]. By definition, it has a higher platelet concentration than what is considered to be physiologically normal. Platelets have been found to affect various biological processes, including angiogenesis, inflammation, cell proliferation, and stem cell migration [13]. In a way, they play a crucial role in initiating the healing process, as they release growth factors that stimulate tissue repair and regeneration [14, 15]. Recent systematic reviews and meta-analyses have reported that the use of PRP as a nonsurgical treatment for patients with PTRCTs could improve shoulder function, reduce pain, and decrease the rate of retearing [16–18].

Vitamin C plays a crucial role in collagen production within connective tissue and bone, and it has been linked to enhanced regeneration of collagen fibers and consequent tendon recovery [19, 20]. It additionally counteracts free radicals, leading to a reduction in inflammation and oxidative stress [21]. Vitamin C promotes the healing of damaged tendons through the proliferation of fibroblasts, increases the diameter of collagen fibers, and enhances local angiogenesis [22, 23]. Additionally, Oakes et al. recently conducted a review in which they suggested that laboratory-animal studies demonstrate favorable outcomes in favor of vitamin C use to accelerate tendon healing [24]. While laboratory research has reported that vitamin C has positive effects on collagen fiber formation and cellular differentiation [25–27], debates persist regarding its effectiveness as a supplement in clinical therapy and further clinical trials are proposed to evaluate the efficacy of vitamin C.

Currently, despite advances in diagnosis and treatment, there is no consensus on the optimal treatment for the management of PTRCTs. As far as we have reviewed, few studies have evaluated the combined treatment of PRP with other drugs for PTRCTs, and the combined therapeutic effect of vitamin C and PRP on tendon repair has not been comprehensively evaluated through rigorous clinical studies to date. The aim of the present study was to evaluate the impact of ultrasound-guided injections of PRP alone and a combination of PRP and vitamin C into the subacromial space on PTRCTs.

## Materials and methods

### Study design

The present study was carried out as a single-center, double-blind, randomized controlled trial. Prior to the intervention, ethical approval was obtained from the Research Ethics Committees of the School of Public Health, Shahid Sadoughi University of Medical Sciences, Yazd, Iran. Moreover, the trial protocol has been registered with the Iranian Registry of Clinical Trials (IRCT), a member of international centers approved by the World Health Organization, and the clinical trial registration code is: *IRCT20230821059205N1.*

The study included patients who were diagnosed with PTRCTs through clinical examination conducted by an orthopedic specialist and whose findings were confirmed through magnetic resonance imaging (MRI). The inclusion criteria for participants were: (1) men and women between 18 and 70 years old; (2) within 6 months of initial diagnosis; and (3) patients with PTRCTs located in the supraspinatus tendon diagnosed via MRI. The exclusion criteria were as follows: (1) pregnant patients; (2) elderly patients over 70 years of age; (3) RCT following fracture and trauma; (4) presence of active infection; (5) history of revision arthroscopy or previous reconstructive surgery; (6) history of PRP injection or other drug interventions, including intra-articular corticosteroid injection; (7) taking anticoagulants within 10 days before intervention; (8) use of NSAIDs 3 days before intervention; (9) history of mental disorders, alcohol, and drug addiction; (10) cardiovascular diseases, diabetes, hemophilia, rheumatoid arthritis, and platelet dysfunctions; (11) shoulder pain mediated by non-rotator cuff tear; (12) platelets < 150,000/µL or hemoglobin < 7 g/dl.

### Patient allocation

All patients who met the inclusion criteria were included in the study after providing informed consent. To evaluate the anterior-posterior tear size, scores 1 and 2 were considered in the MRI findings (score 1, less than 5 mm, and score 2, 5–10 mm) [28].

**Fig. 1:**
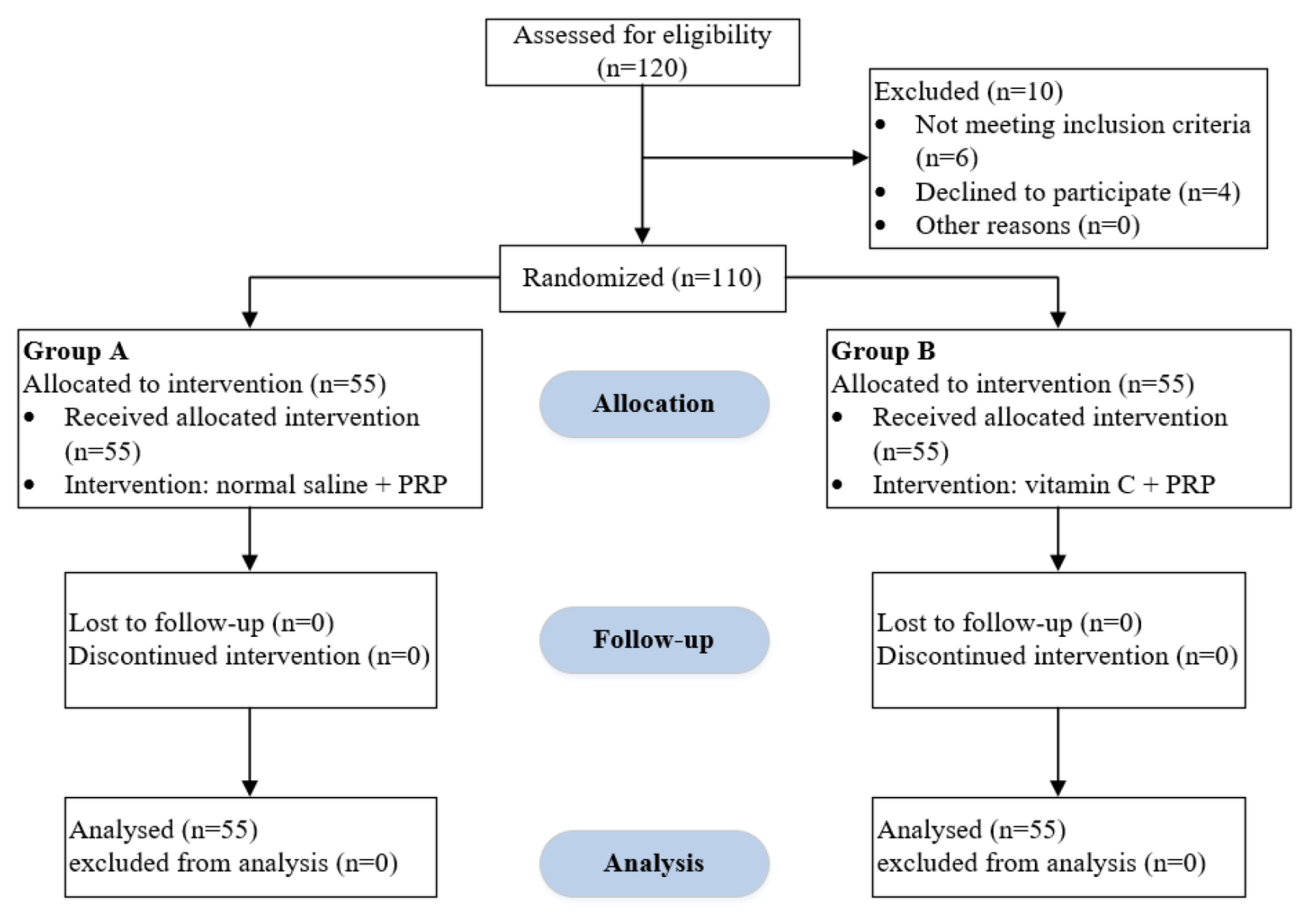
Flow chart of the patient enrollment process. *Abbreviations: PRP platelet-rich plasma*,* n number of patients*

The participants were randomly divided into two groups, A, normal saline + PRP, and B, vitamin C + PRP, by a simple random method and simple random tables created by a computer (Fig. 1). The injection of the therapeutic compound was performed under ultrasound guidance in the subacromial space for two consecutive periods at intervals of 3 weeks. Notably, both injections were performed by the same orthopedic specialist and via the same injection method. Group A was treated with 1.5 ml of normal saline and 1.5 ml of PRP, and group B received 1.5 ml of vitamin C and 1.5 ml of PRP. The study participants, the specialist physician, and the person evaluating the treatment outcome were kept blind during the study. The enrollment, follow-up, and analysis processes based on CONSORT guidelines are displayed in Fig. 1.

### Outcome measures

To assess the level of pain, a visual analogue scale [5] ranging from 0 indicating no pain to 10 (indicating severe pain) was used. The American Shoulder and Elbow Surgeons (ASES) questionnaire, which has items to assess pain and instability and 10 items to measure the patient’s ability to perform activities of daily living (ADL), was used. Additionally, the Constant score was used to evaluate the range of motion and strength of the injured shoulder. To establish baseline scores, a primary assessment was conducted prior to the intervention. Also, the VAS score, ASES score, and Constant score were evaluated 1 and 3 months after the second injection.

### Intervention

To prepare PRP, a 20 ml venous blood sample was collected from each patient and injected into a tube containing an anticoagulant (sterile sodium citrate tubes). The blood was then centrifuged at 1500 rpm for 10 min. Following centrifugation, the plasma located above the red blood cells was carefully transferred to a new sterile tube. The second round of centrifugation lasted for 10 min at a speed of 2500 rpm, and the supernatant (approximately 5–6 ml) was removed so that about 3 ml of plasma along with the cell sediments remained at the bottom of the tube. In the next step, through vortexing, we made the platelet suspension in the remaining plasma in the form of a suspension with an approximate concentration of 5 times the platelet baseline and used it with or without vitamin C for injection into the patients. According to the study by Dhurat and Sukesh [29], this method of PRP preparation is reported as a standard method. In addition, it should be noted that, according to the study conducted by Giacomo et al. [30], the therapeutic dose of vitamin C was considered to be 50 mg/ml.

The intervention was carried out by providing sufficient explanations regarding the objectives of the study, how to perform the intervention, possible side effects, and obtaining informed consent from the patients. After performing a sterile standard preparation, access to the subacromial space was established using an angioket under ultrasound guidance; following this, a sterile syringe containing the therapeutic combination of either normal saline + PRP or vitamin C + PRP was injected into the subacromial space. Eventually, the injection site was disinfected and bandaged. To ensure the absence of possible complications, the patients were monitored for 1 h after the subacromial injection, advised to rest during the first 48 h after the injection, and were also prohibited from using NSAIDs for 1 week.

### Follow up

Patients underwent follow-up at 1 month and 3 months after the second injection; the questionnaires were completed, and the clinical examination was performed by an orthopedic specialist.

### Sample size

The sample size was calculated according to previous studies [31]. Considering the confidence interval at 95% and a power of study of 80% and assuming that the effect size is 0.7, the sample size was calculated to be 55 people for each group for a total of 110 people.

### Statistical analysis

Data analysis was performed using SPSS V.26 The Shapiro‒Wilk test was used to assess the normality of the data. Then, considering that the study data followed a normal distribution, parametric statistical tests were used for evaluation. A chi-square test was used to evaluate the qualitative variables of the study, and quantitative variables were analyzed with a t-test. Repeated measures ANOVA was used to compare the effects of the two treatments on the VAS, ASES and Constant scores.

## Results

A total of 120 patients were screened, of whom 4 refused to enter the study and 6 did not meet the criteria for entering the study. The 3-month follow-up was successfully conducted, and no patients were lost to follow-up. As shown in Table 1, by comparing the demographic characteristics of the two study groups, no significant differences were observed in terms of age, sex, affected side, or dominant limb between the two groups.

**Table 1:** Characteristics of study participants

Variables	Groups A (normal saline + PRP)	B (vitamin C + PRP)	*p*-value
Age, years, mean ± SD	42.87 ± 12.034	43.05 ± 11.621	0.778
Gender (%)			
Male	21 (38/2)	29 (52/7)	0.126
Female	34 (61/8)	26 (47/3)	
Affected side (%)			
Right	30 (54/5)	33 (60/0)	0.563
Left	25 (45/5)	22 (40/0)	
Dominant limb (%)			
Right	36 (65/5)	37 (67/3)	0.840
Left	19 (34/5)	18 (32/7)	

*Abbreviations: PRP platelet-rich plasma*,* SD standard deviations*

Following the assessment of the interaction effects of time and treatment using ANOVA with repeated measures and its nonsignificance (*p*-value = 0.736), the analysis proceeded to the next step. The results of the primary analysis of the VAS, ASES, and Constant scores of the two groups at three time points are shown in Table 2.

**Table 2:** VAS, ASES, and constant scores

	Groups A (normal saline + PRP)	B (vitamin C + PRP)	*p*-value
VAS, mean ± SD			
Pretreatment	7.82 ± 1.827	7.67 ± 1.944	
1 month	5.45 ± 1.476	5.15 ± 1.649	0.389
3 month	3.44 ± 2.053	3.15 ± 1.820	
ASES, mean ± SD			
Pretreatment	33.20 ± 16.811	32.76 ± 16.637	
1 month	53.40 ± 12.125	55.93 ± 12.249	0.578
3 month	71.24 ± 14.619	73.05 ± 12.620	
Constant score, mean ± SD			
Pretreatment	62.40 ± 12.316	61.82 ± 12.214	
1 month	74.15 ± 9.704	74.11 ± 9.183	0.771
3 month	85.02 ± 9.400	87.13 ± 6.804	

*Abbreviations: PRP platelet-rich plasma*,* SD standard deviations*,* VAS Visual Analogue Scale*,* ASES American Shoulder and Elbow Surgeons*

**Fig. 2:**
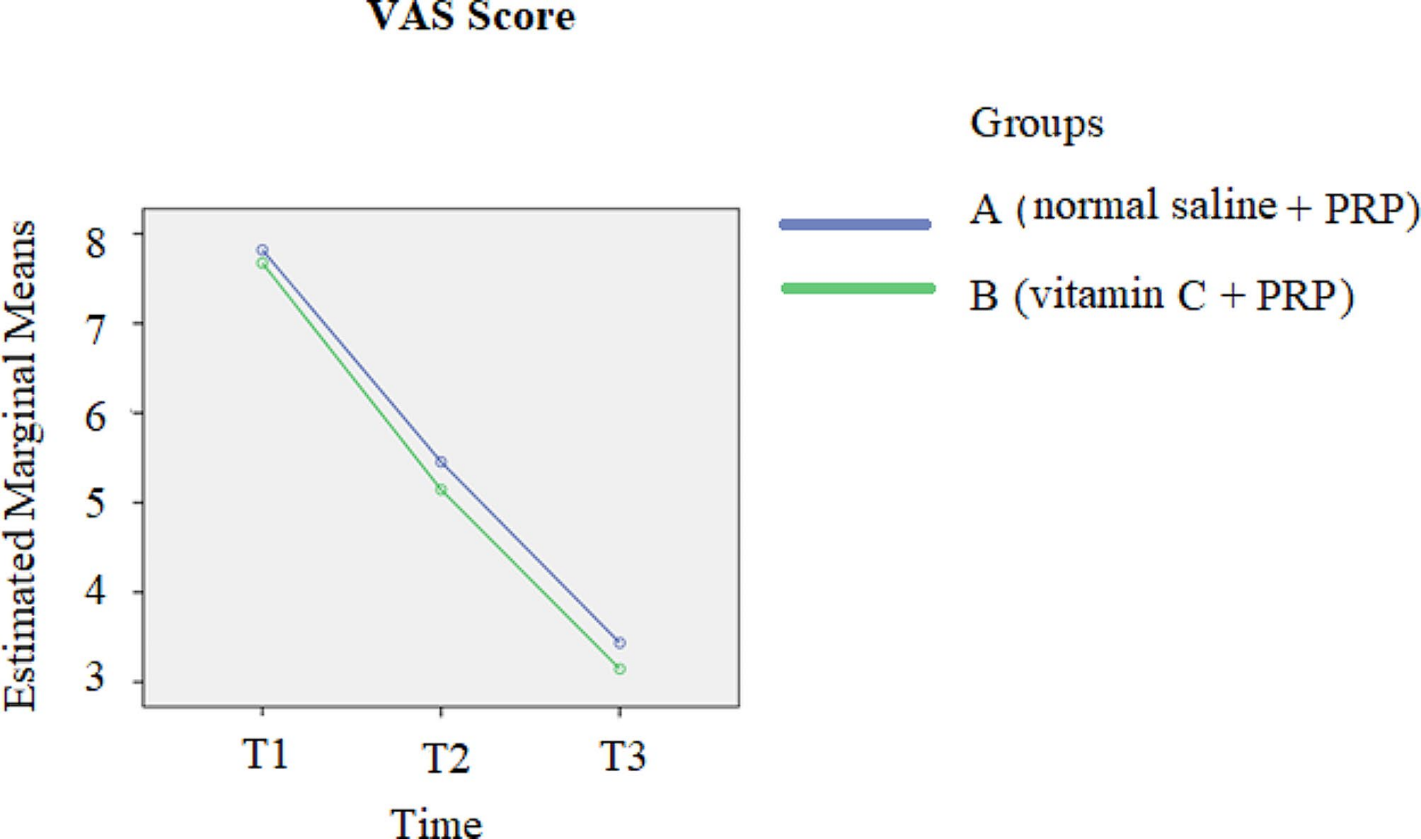
Comparison of the VAS scores between the two groups at three time points T1: pretreatment; T2: 1 month after treatment; T3: 3 months after treatment. *Abbreviations: PRP platelet-rich plasma*,* VAS Visual Analogue Scale*

**Fig. 3:**
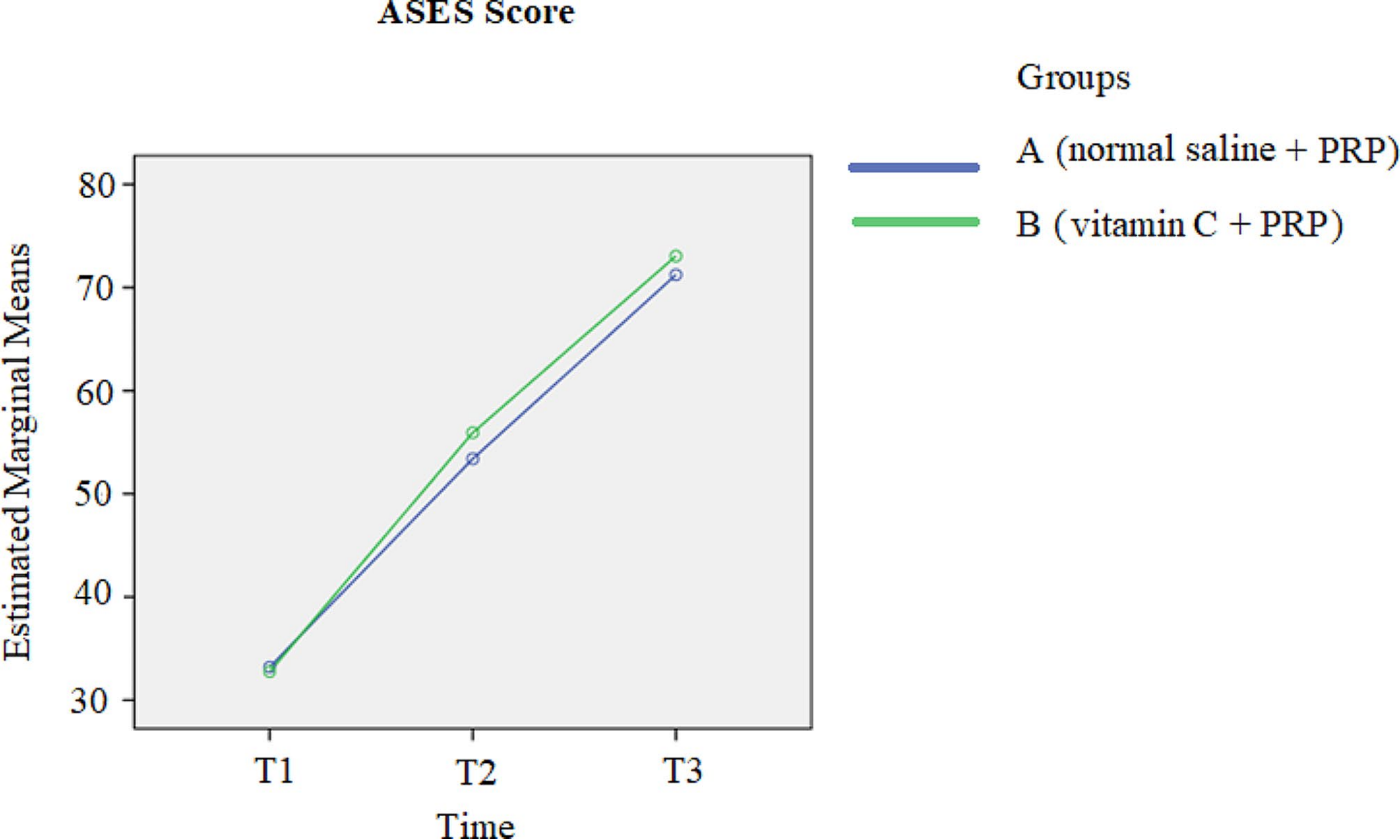
Comparison of the ASES scores between the two groups at three time points T1: pretreatment; T2: 1 month after treatment; T3: 3 months after treatment. *Abbreviations: PRP platelet-rich plasma*,* ASES American Shoulder and Elbow Surgeons*

**Fig. 4:**
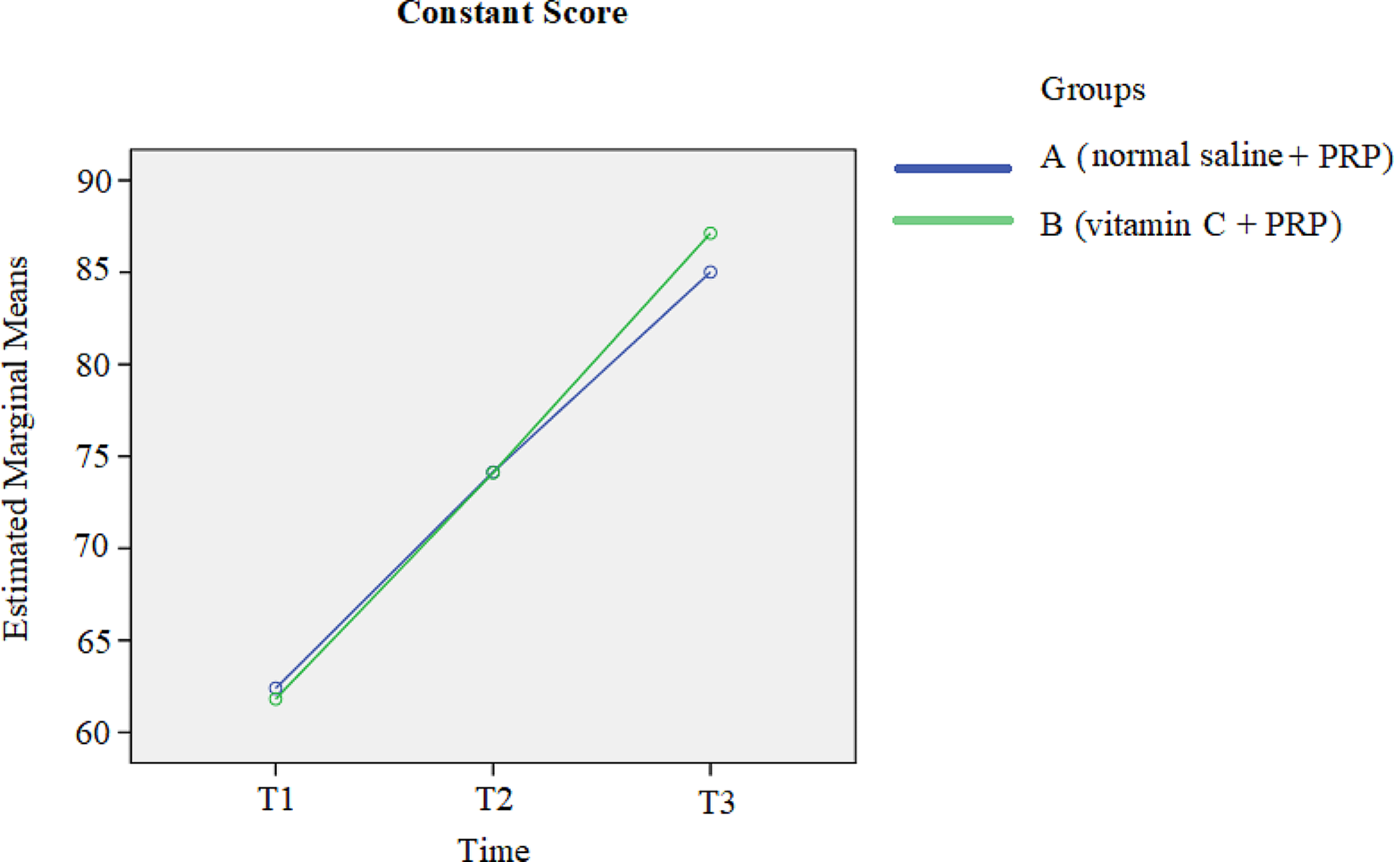
Comparison of the Constant scores between the two groups at three time points T1: pretreatment; T2: 1 month after treatment; T3: 3 months after treatment. *Abbreviations: PRP platelet-rich plasma*

A comparison of the pain scores between the two groups at the 3-month follow-up revealed a reduction in pain in both groups over time (*p*-value < 0.001). Additionally, shown in Fig. 2, group B exhibited greater pain reduction than did the other group; however, this difference was not statistically significant (*p*-value = 0.389). Figure 3 shows that the ASES scores of both groups increased over time (*p*-value < 0.001); moreover, in the comparison of the ASES scores between the two groups at follow-up points, a further increase in ASES score was observed in group B compared with the other group, while this difference was not significant (*p*-value = 0.578). The comparison of the Constant scores between the two groups at three time points revealed that at one month following the treatment, the results were completely the same. After 3-months of treatment, despite the increase in Constant score in favor of group B, no statistically significant difference was found between the two groups (*p*-value = 0.771). Similarly to the other scores, the Constant scores of both groups increased during the 3-month follow-up (*p*-value < 0.001). More details are highlighted in Fig. 4.

## Discussion

The results of the current study demonstrated that both PRP alone and PRP combined with vitamin C could be considered promising treatments for patients with PTRCTs. However, contrary to expectations, the present study did not find a significant difference between the two groups, neither in pain reduction nor in ASES and Constant scores promotion. This finding is consistent with the study of Martel et al. [32], who aimed to explore how administering vitamin C supplementation after surgery affects tendon healing. They reported that at the 6-month follow-up, the difference between the two groups was not significant; nevertheless, the rate of nonhealing was greater in the group that did not receive vitamin C than in the group that had been treated with vitamin C [32]. In an animal study, Turkmen et al. investigated the effect of a Mucopolygen Complex (containing mucopolysaccharides, hydrolyzed collagen type I, and vitamin C) on Achilles tendon rupture. While the intervention group showed more regular collagen formation compared to the control group, there was no significant improvement in the mean tolerable load of the Achilles tendon before injury [33].

Uehara et al. studied the effect of antioxidants (vitamin C and NAC) on rotator cuff healing in rats. Rats underwent bilateral tendon repair surgery and were divided into three groups. The study found that both NAC and vitamin C reduced oxidative stress and accelerated healing [27].

Moreover, another study by Morikawa et al. showed that vitamin C, through its antioxidant effect, diminished degeneration of the rotator cuff in the same way as that observed in humans and in mice with vital antioxidant enzyme deficiency [34]. Similarly, Giacomo et al., investigated the impact of ascorbic acid on cell survival and proliferation. They specifically focused on cells derived from human tendons and examined the effects of combining ascorbic acid with the thyroid hormone T3 in an in vitro model, and concluded that ascorbic acid is an inducer of cell proliferation that causes tenocyte growth, whereas it decreases nitric oxide synthesis [30]. The synergistic effect of vitamin C and growth factors was also reported in an animal study [35]. A possible explanation for these contrary outcomes to our findings might be the difference in the design of clinical and laboratory studies and the different dosages of vitamin C administered. In addition, this inconsistency may also be explained by the recent systematic review regarding the effectiveness of vitamin C supplementation on oxidative stress and collagen synthesis following orthopedic injuries, which noted that clinical data do not reduplicate the results reported in animal studies at present [36].

As discussed earlier, within the 3-month follow-up, it was observed in both groups that the patients’ pain was alleviated significantly, and regarding the ASES and Constant scores, an increase in the patients’ scores was evident. These results are consistent with those of Dadgostar et al. (2021), who also reported that PRP injection significantly reduced pain and improved the range of motion at the 3-month follow-up [37]. Similarly, the positive effect of PRP in improving function and relieving pain in patients with tendinopathy and PTRCTs was highlighted by Kwong et al. [38]; these findings are consistent with recent clinical observations [39–41]. On the other hand, this outcome is inconsistent with that of Schwitzguebel et al. [42], who did not report superiority in terms of pain improvement and clinical scores for the intervention group compared to the control group after PRP injection within interstitial supraspinatus lesions. The unavailability of clinical results in the short-term phase and the fact that only treatment was evaluated 7 months postintervention may be limitations of this study. Additionally, Carr et al. [43], reported similar results regarding the inefficiency of PRP treatment. These conflicting results could be associated with differences in injection site and method, different methods of PRP preparation, dissimilarity in research design, and differences in tendon tear size among various studies.

No complications or side effects were observed following the intervention; as reported, low doses of vitamin C are associated with minor side effects [21]. The ASES score has items related to evaluating the activities of daily living; the increase in the ASES score of both groups over time refers to the enhancement of the activities of daily living. Similarly, the improvement in participants’ shoulder strength and range of motion following the increase in the Constant score within 3 months was significant.

The evaluation of tendon tear size changes with MRI requires a follow-up of more than 6 months, while the follow-up intervals in the present study were 1 and 3 months; hence, the final evaluation of the patients was carried out by a specialist.

The present study was limited in several aspects. First, the study focused on evaluating the treatment group that received the combined injection of PRP and vitamin C. While the inclusion of a control group that received either no injection or a sham (placebo) injection could have enabled a more robust comparison of outcomes. Another limitation was the absence of radiological assessments, such as ultrasound or MRI, between the treatment and control groups at baseline and during follow-up. Incorporating these objective measures would have provided valuable data on changes in the studied condition over time, enabling a more comprehensive evaluation of the treatment’s effectiveness. An additional limitation is the relatively short follow-up period. Following the same group of participants for a longer duration and including MRI outcomes at the end of the follow-up would have provided valuable insights into the long-term effects of the treatment. However, the results have shown that the outcome obtained from nonsurgical treatment in patients with chronic RCTs at 3 months is predictive of the outcome 2 years after treatment [44].

Furthermore, the present research was limited by several other factors, including a lack of consensus regarding the effective frequency of PRP injection, an uncertain optimal dosage and concentration of vitamin C and PRP. Moreover, as far as we know, the present research is the first controlled clinical trial that investigated the effect of combined injection of PRP and vitamin C in the subacromial space in patients with PTRCTs; therefore, the limited number of previous clinical studies was one of the limitations of comparing the results of this study with those of similar studies.

In terms of directions, future prospective clinical studies should aim to include a wider patient population, incorporate radiological assessments, and extend the follow-up period to offer valuable insights into the long-term effects and durability of the treatment. Conducting studies with these design elements would help to further elucidate the effectiveness of combined vitamin C and PRP injections on tendon healing.

## Conclusion

In conclusion, our study demonstrates that both PRP alone and PRP combined with vitamin C can effectively reduce pain and improve function in patients with PTRCTs. Notably, both interventions resulted in significant improvements in patient outcomes, with no significant difference between the two groups at 3 months of follow-up. Related study topics will be a fruitful area for further research, and we plan to continue this study by evaluating MRI findings over a longer follow-up period.

## Data Availability

No datasets were generated or analysed during the current study.

## Abbreviations

ADL: Activities of Daily Living
ASES: American Shoulder and Elbow Surgeons
RCT: Rotator Cuff Tear
ROM: Range of motion
NSAID: Nonsteroidal anti-inflammatory drug
PRP: Platelet Rich Plasma
PTRCT: Partial Thickness Rotator Cuff Tear
VAS: Visual Analog Scale
